# How has publishing changed in the last twenty years?

**DOI:** 10.1098/rsnr.2016.0035

**Published:** 2016-08-31

**Authors:** Sunetra Gupta

**Affiliations:** Department of Zoology, University of Oxford, The Tinbergen Building, South Parks Road, Oxford OX1 3PS, UK

**Figure 1.**
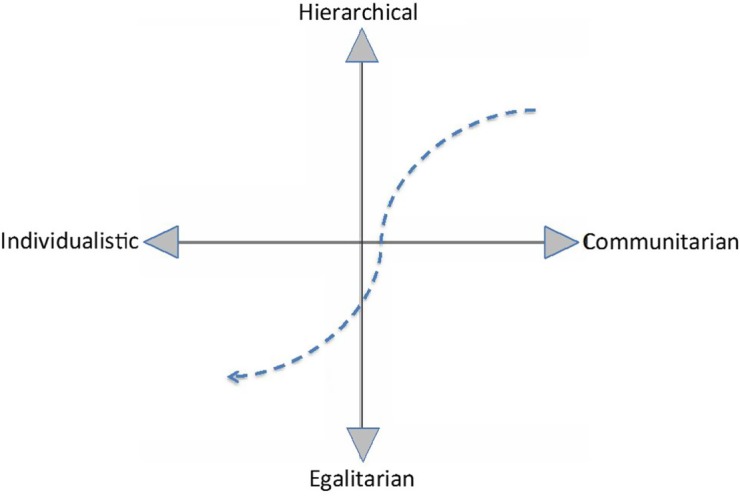
The trajectory of scientific and literary publishing within ‘grid-group’ space since the 1990s. (Online version in colour.)

It is useful to consider the trajectory of both scientific and literary publishing on the grid-group plane defined by Mary Douglas which arranges attitudes along two axes: one ranging from the hierarchical to the egalitarian, and the other spanning individualistic to communitarian (figure 1). I would contend that, in both cases, there has been a move from the hierarchical/communitarian quadrant towards the egalitarian/individualistic zone. This is probably a reflection of the drift towards neo-liberalism in academia and society at large, and shares some of its basic features, namely (i) an emphasis on a ‘finished’ product of obvious immediate utility, (ii) intense competition among peers, resulting in vicious, irrational reviewing of scientific papers, and the promotion of ‘cartels’ among the scientific community. The overall result, naturally, is a constant choke on the production of new ideas which are (i) yet to be supported by evidence and therefore have low market value, and (ii) much more likely to destabilize the careers of individuals within the competitive neo-liberal framework than under the somewhat feudal system it has replaced.

Stipulating that new ideas have to be consummately validated (rather than simply being rigorously constructed) before they can be presented to a general audience actively discourages the creation of new hypotheses. Indeed, most of science as it is practised now is built upon a set of hypotheses for which there was very little ‘compelling evidence’ at the time they were published. Natural selection, the germ theory of disease, the Higgs boson—all existed as concepts before they came to be observed. The current system, therefore, fundamentally discriminates against the process of hypothesis generation, which is as integral to science as hypothesis testing. The emphasis on ‘compelling evidence’ for a theory echoes the insistence of the literary publishing industry for complete and well-researched narratives: both are fundamentally anti-experimental stances. Indeed by refusing to consider a (rigorously formulated) hypothesis before it has been fully validated, the scientific publishing industry may—in effect—cut off the means by which it may be tested, since this can only be achieved through the highly competitive process of obtaining research funding where the chances of success are tightly linked to the applicant's publication record. Unless we intervene, we are at risk of causing untold harm to a generation of young people who were attracted to science precisely because of its potential to transform how we think; conducting science by these neo-liberal precepts ultimately poses a danger to society. It remains to be seen whether the publishing industry will continue to adapt to, and capitalize upon, an increasingly market-driven approach to science, or turn away from it and open up new spaces for creative ideas to flourish.

